# Gamma Irradiation of Hexafluorobenzene^1^

**DOI:** 10.6028/jres.064A.026

**Published:** 1960-08-01

**Authors:** R. E. Florin, L. A. Wall, D. W. Brown

## Abstract

Mixtures of hexafluorobenzene and benzene were irradiated in liquid phase by means of a Co^60^ gamma source at 20° and at 218° C. Perfluoroheptane and various binary mixtures involving perfluoroheptane, hexafluorobenzene, benzene, and cyclohexane were also irradiated at 20° C. Hexafluorobenzene resembled benzene very closely in its behavior upon radiolysis. Generally the fluorocarbon-hydrocarbon mixtures evolved much more SiF_4_ (indicating the formation of HF, which reacts with the glass vessel) than the pure fluorocarbon components. The polymer from hexafluorobenzene-benzene mixtures was probably rich in cyclohexadiene and cyclohexene units, resembling that from pure benzene, and its composition ratio exhibited a strong “alternating” tendency. The results are discussed in terms of free-radical and excited-state mechanisms. At 218° C hexafluorobenzene and also its mixtures with benzene showed qualitative differences from their behavior at 20° C, although the *G* values for SiF_4_ and polymer remained moderate.

## 1. Introduction

Fully fluorinated aromatic compounds have only recently become accessible. Because of their combination of C—F bonds and aromatic resonance structure they have attracted interest as possible heat-resistant materials. It has been observed that certain polyphenyls (C_6_F_4_)*_x_* are stable at high temperatures [1]^2^ and that the mass spectrum of these compounds shows relatively little fragmentation under electron impact [2]. The latter observation is reminiscent of benzene itself, and contrasts strongly with the extensive fragmentation of saturated fluorocarbons in the mass spectrometer [3,4].

Since electrons are an important intermediate in the action of ionizing radiation, it can be expected that hexafluorobenzene derivatives, like their hydrocarbon analogs, will be relatively resistant to radiation, and may surpass them under some combinations of high temperature and radiation. Any improvement in materials for use under such conditions would be desirable.

The radiation chemistry of fluorocarbons has been studied very little until very recently, except for a few polymers and monomers [5 to 14]. Halogen compounds, including a few monofluoro derivatives, have been investigated and generally have been found to exhibit a very high sensitivity to radiation [15]. Benzene has been studied very extensively, both because of its low sensitivity and the possibility that it can exhibit a “protective” effect in mixtures. Likewise, a few higher aromatic hydrocarbons have been studied [16 to 18]. In radiation chemical studies in general, atom and free-radical mechanisms have been fairly well accepted [19], though seldom unequivocally proven. Besides the great wealth of data on hydrocarbon radical reactions [20], there have recently been a number of studies on reactions of aliphatic fluorocarbon radical [21 to 29].

The present situation is that fluorocarbons seem more resistant than hydrocarbons to heat, and also aromatic compounds more resistant than aliphatic. Under ionizing radiation, aliphatic hydrocarbons give off relatively large amounts of hydrogen; aromatic hydrocarbons lose very little hydrogen, but form polymers in moderate yield. Aliphatic fluorocarbons suffer breaks in the carbon chain and lose slight to moderate amounts of fluorine in ionic form [7 to 9]. The aromatic hydrocarbons biphenyl and terphenyl are resistant enough to radiation at high temperatures to be of some interest as reactor coolants [18]. The order of magnitude of the radiation yield, *G*, in molecules per 100 ev absorbed, is indicated in table 1 for the several processes. In this paper, we report some observations on the irradiation of C_6_F_6_, C_7_F_16_, and their mixtures with other materials.

## 2. Experimental Procedure

In general, samples were prepared on a vacuum line and irradiated to doses of about 100 to 300 megaroentgens (Mr). Products volatile at −80° and 25° C were analyzed by mass spectrometer. Nonvolatile residues were isolated by distillation of the liquid and characterized by infrared spectra and elemental analysis. The hexafluorobenzene used was from a specially purified center cut of material synthesized in our laboratory [30] and subjected to repeated fractional freezing. Its constants were bp =80.5° C/759 mm, $n_{23}^{D}=1.3781$. The benzene used was the commercial product (c.p.), not further purified. The perfluoroheptane used was the product of Minnesota Mining & Manufacturing Co. and was used as received. The cyclohexane used was an NBS standard sample.

For the irradiations at room temperature, the liquids were dried several weeks over P_2_O_5_ in break-seal tubes and distilled on the vacuum line into weighing bulbs of 30-ml capacity. These bulbs were fitted with standard taper joints and Hoke bellows valves connected via copper or Kovar glass seals. Samples were made up by distilling *in vacuo* into irradiation vessels of several kinds, then degassing, cooling with liquid nitrogen, opening for about 1 minute to a measured pressure of a few centimeters of argon, and sealing or closing off. Weights of components of the mixture, obtained by difference, were checked against the total weight of the radiation vessel. Discrepancies of a few milligrams were common, probably because of absorption of the volatile liquids in stopcock grease.

The radiation vessels for use at 218° C were 3-mm thick-walled glass tubes. At room temperature nickel capsules of about 10- to 15-ml capacity were used. They were silver-soldered to 1.5-mm × 40-mm nickel tubes and then through 4.7-mm o.d. Kovar seals to long 3-mm o.d. Pyrex end sections which were designed for opening into the mass spectrometer inlet system. In one irradiation experiment under a high pressure of hydrogen, a third type of radiation vessel was used, consisting of a Monel bomb, 12-mm o.d. × 6-mrn i.d. × 300 mm in length, closed by a Hoke bellows valve. The simple capillary tube had the advantages of strength and small size but had a large potentially reactive wall surface and also could not be opened for analysis without some exposure to air. The composite capsules were mostly corrosion resistant, vacuumtight, and easily fitted to the mass spectrometer but were very fragile after use. The existence of a partial internal glass surface had some advantages as well as drawbacks. Any HF formed was converted to the easily measured SiF_4_, while any reactive fluorocarbon intermediates of short life probably underwent further reaction in the liquid system before they could diffuse to the glass surface. It was noticed that corrosion was especially severe in the special graded glass of the Kovar seal. The Monel bomb vessel had the advantage of strength and all-metal construction but probably was subject to slow leaks over long periods. It is uncertain whether it would have remained tight with the valve exposed to high temperatures.

The sample tubes to be irradiated were placed in a can and lowered into the uniform central region of a 2,000-curie cobalt-60 source consisting of upright rods arranged in a circle and shielded by water. Heat, when needed, was provided within the can by a thermostat furnace capable of reaching 500° C. Electrical leads were carried through a pipe leading to the surface of the water. The exposure dose rate was determined by the ferrous sulfate dosimeter using *G*= 15.5, and by a time correction for decay of the cobalt. The dose rate was 0.576 Mr/hr on December 11, 1956. The variations with geometry (mainly vertical extension) and container wall shielding were significant only for the Monel bomb containers. The absorbed dose was calculated with the aid of best values for the elements, derived from ref [31]. Typical factors in ev g^−1^ Mr^−1^ ×10^−20^ were: C_n_H_2n_, 0.623; C_6_H_6_, 0.589; C_n_F_2n_, 0.526; and C_6_F_6_, 0.530.

After irradiation, which required several weeks, the samples were brought to the mass spectrometer whenever the design of the vessel was appropriate, and mass spectra taken with contents at −80° and +25° C. Where the container was not adapted for this, the contents were first transferred on an auxiliary vacuum line. Many of the Kovar-seal containers in which hydrocarbon-fluorocarbon mixtures were exposed to high doses were broken during irradiation or subsequent handling because of corrosion at the glass-metal seals. To calculate the mass spectrometer results for gaseous products, reliance was placed upon the argon introduced as an internal standard. For liquid products, the examination at 25° C yielded only relative values, distorted by fractionation effects. The opened tubes were emptied by suction, and the liquid content was frozen and sublimed at reduced pressure to isolate the nonvolatile residue. The empty tubes were rinsed with benzene repeatedly, yielding small additional amounts of residue. The residues were analyzed^3^ for C, H, and F, and infrared spectra^4^ were also taken in films and Nujol mulls.

In the mass spectrometric procedure, bulbs containing a large liquid sample were connected to the inlet system of the mass spectrometer before breaking the seal. Analyses of volatiles were then made with the sample bulb first cooled to −80° C and then warmed to 25° C. The analysis of material volatile at −80° C should give a reasonably accurate estimate of gaseous products for all samples in which the liquid was completely frozen at that temperature. This includes all samples containing hexafluorobenzene, benzene, and cyclohexane only. However, perfluoroheptane, although the reported freezing point is −55°, is often still liquid at −80° C and therefore can hold large quantities of gases in solution. For samples containing perfluoroheptane, therefore, the mass spectrometric analyses at −80° C may seriously underestimate the yields of gases. This error will be greatest for the samples rich in perfluoroheptane and for the higher boiling gases such as CF_4_ (bp −128° C), SiF_4_ (subl −95° C), C_2_F_6_ (bp −76.3° C), and CF_3_H (bp −84.4° C).

For similar reasons, the volatiles at 25° C have merely qualitative interest. Products less volatile than the starting material will appear in greatly reduced concentration. The results will be especially uncertain for products of intermediate volatility, which are depleted in supply because of the previous analysis at −80° C, but favored by high relative volatility at 25° C.

Polymeric residues in irradiated samples were analyzed by combustion methods. In many instances, the sum of C, H, and F is low by several percent. The deficit may be attributed either to poor accuracy of fluorine analyses or to oxygen absorbed during the period between opening of samples and analysis. The polymeric product from irradiated benzene is highly reactive with oxygen [16], and the related material from fluorocarbons could react not only with oxygen but with moisture as well. These reactions should have been minimized in the present work by the fact that the longest storage of samples was in the crystalline or glassy form. It will therefore be assumed in calculations that the total deficit is due to low fluorine analyses.

A nearly self-consistent account can be given of the composition of C_6_F_6_—C_6_H_6_ residues by postulating combination of molecules and elimination of HF. The calculations will be deferred until the discussion of these mixtures. In most other mixtures, the polymer analysis is consistent with several possibilities, but extremes can be calculated.

The metallic interior surfaces of the containers appeared unchanged after irradiation. A monomolecular layer of metal fluoride may possibly have been present, but this could not contribute important errors at the doses used in this work.

All of the systems containing some fluorocarbon and some glass produced SiF_4_.

In the capillary tubes containing C_6_F_6_ at 218° C, this product may have been formed by direct reaction of excited molecules with the wall. In the composite metal bulb reactors, the long diffusion path makes it very unlikely that short-lived intermediates of any kind could reach the glass parts in significant quantity. Among possible agents attacking the glass are F_2_, HF, and perhaps especially reactive fluorocarbon molecules.

Radicals or fluorocarbon molecules attacking glass should produce CO or CO_2_, as well as SiF_4_:

$$4{\text{CF}}_{3}\cdot +3{\text{SiO}}_{2}\to 3{\text{SiF}}_{4}+2\text{CO}+2{\text{CO}}_{2}.$$ (1)

The steps would involve gradual replacement of O by F in the glass lattice until a volatile SiF_4_ molecule is produced:

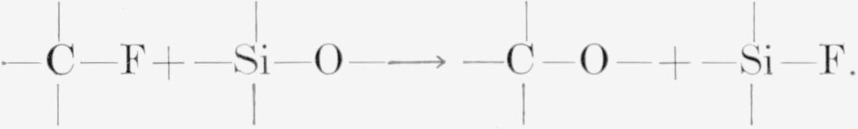
(2)Reactions not producing oxides of carbon are also possible:

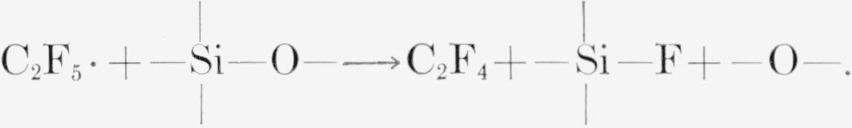
(3)The reaction of F_2_ with glass would apparently produce oxygen as a byproduct:

$$2F_{2}+{\text{SiO}}_{2}\to {\text{SiF}}_{4}+O_{2}.$$ (4)

This oxygen could react with radicals to form oxides of carbon or oxygenated fluorocarbon compounds. Despite the known slowness of the glass-fluorine reaction, it appears likely that it should occur in appreciable amounts over the long radiation times. Even in view of the uncertainties implied by the above reactions, it seems reasonable to consider each SiF_4_ molecule as derived from 4 HF in mixtures with hydrocarbons, and from 2F_2_ in pure fluorocarbon systems, unless equivalent amounts of CO or CO_2_ are observed. The CO and CO_2_ were usually observed only in very small amounts relative to SiF_4_. The reaction involving HF should produce water as a by-product:

$$4\text{HF}+{\text{SiO}}_{2}\to 2H_{2}O+{\text{SiF}}_{4}.$$ (5)

Conversion of HF to SiF_4_ is presumed to be nearly complete, although some fixation in the form of alkali fluosilicates is conceivable. The above reactions show that the later stages of the irradiation may be complicated by gradually increasing amounts of oxygen and water.

Most of the materials were irradiated only at room temperature, but hexafluorobenzene and the C_6_F_6_–C_6_H_6_ mixtures were irradiated at both 20° and 218° C.

## 3. Results and Discussion

### 3.1. Hexafluorobenzene

From pure hexafluorobenzene the observed products were a nonvolatile residue (the so-called “polymer/’ table 2) and SiF_4_ (tables 3 and 4) with a little CO and CO_2_, which may have been derived from fluorine atoms or molecules or unstable fluorocarbon intermediates. At 20° the release of fluorine was almost negligible, *G*(SiF_4_) = 0.01, but at 218° it became 0.21 molecules/100 ev. The yield of polymer was about the same at both temperatures within the large experimental error at 218° and was about twice that from benzene. *G*(polymer) = 2.01 at 20°, 1.3±0.5 at 218°. The character of the polymer changed greatly with temperature, being a light yellow, low-melting (<100°) glass at 20°, and a nearly black finely granular precipitate at 218°. The elemental analysis of the 20° polymer was near that of the parent compound (table 2). The deficit, 100—C—F —H, and H content may represent contaminations in handling, difficulties of quantitative fluorine determination, or in the case of the deficit possibly oxygen absorption during storage [16, 17]. The quantity of the 218° C polymer was not sufficient for analysis.

**Figure 1:**
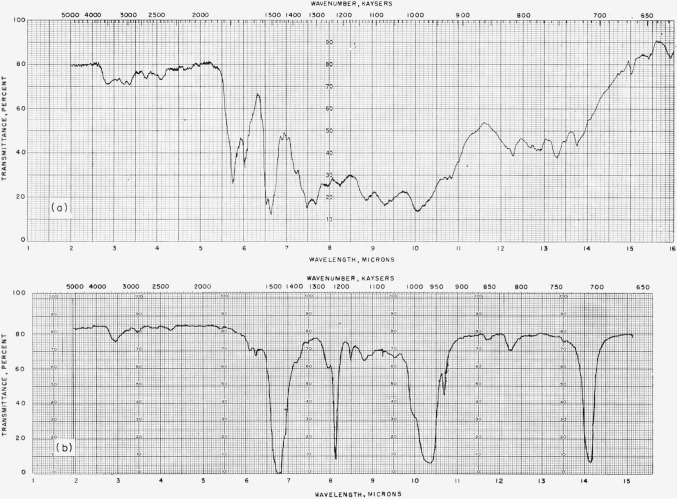
Infrared absorption spectra of hexafluorobenzene radiation polymer and of perfluoropolyphenyl. a. C_6_F_6_ radiation polymer, b. I-(C_6_F_4_)_11_-I.

Infrared spectra of the polymer and a synthetic perfluoropolyphenyl are compared in figure 1. Both have strong peaks at 6.6 and at 10.15 *μ*, but the radiation polymer has a broader absorption generally and numerous additional peaks at 5.7, 7.5, 8.8, 12.8, 13.3, and 13.7 *μ.* Most of the absorption bands are consistent with a C—F bond adjacent to either an aromatic or an olefinic carbon atom. The infrared absorption offers no reliable basis for a distinction.

There appear to be no small fluorocarbon molecules analogous to the C_2_H_2_ and CH_4_ found with benzene. The similarity of C_6_F_6_ to C_6_H_6_ was striking—very low yields of volatile products, and a moderate yield of polymer; *G*(polymer) = 2.01 for C_6_F_6_, and 0.93 for C_6_H_6_.

It is recognized that nearly the total effect of ionizing radiation on organic matter is due to the secondary electrons. Their first effect is to form positive ions which can be important intermediates in the gas phase [32] but are more likely to recapture electrons in liquid phase and form neutral radicals and atoms. Although the number of excited molecules (singlet and triplet) may considerably exceed the number of unexcited radicals formed [19], it is often possible to restrict attention to atoms and radicals as the effective chemical intermediates. Feng [13, 14] has briefly considered ionic intermediates, pointing out that atom formation is energetically less favorable for carbon-fluorine than for other carbon-halogen bonds, and other investigators [16, 17] have introduced excited states in the discussion of the radiolysis of benzene.

The radiolysis of hexafluorobenzene offers few novelties beyond its hydrocarbon analog; both the free-radical mechanisms and excited-state mechanisms [16, 17] seem admissible with little choice. The outlines of a free-radical mechanism, following Burton’s [16] treatment of C_6_H_6_, would be

$$C_{6}F_{6}\to C_{6}F_{5}\cdot +F\cdot ,$$ (6)

$$C_{6}F_{6}+F\cdot \to C_{6}F_{7}\cdot ,$$ (7)

$$R\cdot +\;C_{6}F_{6}\to {\text{RC}}_{6}F_{6}\cdot \left(R\cdot =C_{6}F_{5}\cdot ,C_{6}F_{7}\cdot ,C_{6}F_{6}C_{6}F_{5}\cdot \right),$$ (8)

R⋅+R⋅→RR, (9)

R⋅+F⋅→RF. (10)

Reactions (7) and (8), by analogy with the hydrogen atom-benzene reaction, probably have an activation energy of several kilocalories at most. The activation energy of reaction (8) would be reduced for C_6_F_6_ in an excited state. The low yield (*G*=2.01) does not require a chain reaction; however, the structure of the polymer (less volatile than biphenyl, melting below 100° to a moderately viscous liquid) requires a few addition steps like reaction (8). No abstraction reaction has been introduced:

$$R\cdot +C_{6}F_{6}\to \text{RF}+C_{6}F_{5}.$$ (11)

External evidence against reaction (11) is twofold. In the first place, in hydrocarbon analogs reaction (11) is slower than (8) at room temperature and below, e.g., addition dominates in the photochlorination of benzene [20] and the reaction of H atoms with frozen benzene [33]. The ratio, addition/abstraction, may be about 7.5 in radiolysis of mixtures of C_6_H_6_ and C_6_D_6_ [34].

Observation of C_6_H_5_CF_3_ in the radiolysis of mixtures of CF_4_ and C_6_H_6_ [13, 14] may require abstraction from C_6_H_6_ if the mechanism is of the free-radical type. Abstraction of H from C_6_H_6_ is postulated in the radiolysis of dilute aqueous benzene, the ultimate products being C_6_H_5_OH and (C_6_H_5_)_2_ [35]. In both these instances, the attacking radical is highly electronegative.

The second evidence against reaction (11) is that fluorine atoms are not readily abstracted from perfluoroparaffins by ordinary atoms and radicals such as H [36], CH_3_ [37], CF_3_ [28], and C_2_F_5_ [27, 29]. Presumably, the fluorine atoms of aromatic fluorocarbons are likewise resistant. For the abstraction reaction

$$H\cdot +{\text{CF}}_{4}\to \text{HF}+{\text{CF}}_{3}.$$ (12)

*E*> 17 kcal, and the reaction is not observed up to 400° C [36].

For C_2_F_4_ [38] and C_6_H_5_F [39] reacting with H atoms, the evidence is for an efficient addition rather than abstraction.

In summary, it therefore seems unlikely that fluorine will be abstracted from either C_6_F_6_ or C_7_F_16_, except perhaps by “hot” atoms or radicals. Thus, there remains a radical mechanism with dissociation, addition, and recombination steps, yielding a polymer largely nonaromatic. The very low yield of SiF_4_ requires that C_6_F_6_ should be a very efficient trap for F atoms. If the F atoms are formed in an efficient cage of C_6_F_6_ molecules, reactions (7) and (10) can predominate over reaction (13)

$$F\cdot +F\cdot \to F_{2},$$ (13)

without requiring any great inequality of rate constants.

Gordon and others [17, 40] have written a mechanism for C_6_H_6_ radiolysis involving excited states only. The same mechanism can be written for C_6_F_6_:

$$C_{6}F_{6}\to C_{6}F_{6}*,$$ (14)

$$C_{6}F_{6}*+C_{6}F_{6}\to {\left(C_{6}F_{6}\right)}_{2}.$$ (15)

Hydrocarbon analogs of the dimer have been reported. An advantage of the excited-state mechanism is that the nearly complete absence of SiF_4_ (derived from corrosive fragments) is explained simply when fragments are not formed.

At higher temperatures (218° C) the argument against the radical mechanism does not apply, as considerably more SiF_4_ is formed. The actual behavior of C_6_F_6_ at higher temperatures offers some difficulties. The black granular insoluble polymer suggest a highly condensed aromatic ring structure formed by extensive elimination of fluorine, yet the yield of polymer is about the same as that at low temperature, and the SiF_4_ equals somewhat less than 1 F atom per C_6_F_6_ ring (see table 4).

### 3.2. Hexafluorobenzene and Hydrogen

Hexafluorobenzene and hydrogen produced more SiF_4_ (from HF) than hexafluorobenzene, but less than in mixtures with hydrocarbons (see table 5, cf. tables 3 and 4). The pressure of hydrogen was 34 atm at −80° C, corresponding to 0.0223 mole in the sample, and the amount of hexafluorobenzene was 0.0176 mole. Assuming pertinent properties of the C_6_F_6_ to be the same as those of C_6_H_6_, reasonable estimates for the composition are
*in vapor phase**in liquid phase*C_6_F_6_ 5.67×10^−5^ mole0.0176 moleH_2_ 0.223 mole  .00017 moleThe dose was 319 Mr. Much of the vapor was in a less intense radiation field. For the calculations in table 5, it is assumed that all radiation was absorbed by C_6_F_6_ as liquid.

The value of *G*(SiF_4_) is 0.11 as against 0.01 for pure C_6_F_6_ and 0.33 for a mixture of C_6_F_6_ and C_6_H_6_.

Conceivable steps producing the HF may be:

$$F\cdot +H_{2}\to \text{HF}+H\cdot ,$$ (16)

$$C_{6}F_{5}\cdot +H_{2}\to C_{6}F_{6}H+H\cdot ,$$ (17)

$$C_{6}F_{7}\cdot +H_{2}\to C_{6}F_{7}H+H\cdot ,$$ (18)

$$C_{6}F_{7}\cdot +H_{2}\to C_{6}F_{6}+\text{HF}+H\cdot ,$$ (19)

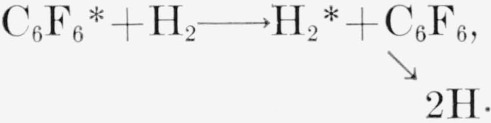
(20)

$$C_{6}F_{6}*+H_{2}\to \text{HF}+H\cdot +C_{6}F_{5}.$$ (21)

These reactions must compete with addition reaction (8) and must be roughly comparable with reactions for HF production in hydrocarbon mixtures, e.g.,

$$F\cdot +C_{6}H_{6}\to \text{HF}+C_{6}H_{5}.$$ (22)

Any H atoms produced in reactions (16) to (20) may react by addition or abstraction:

$$H\cdot +C_{6}F_{6}\to C_{6}F_{6}H\cdot ,$$ (23)

$$H\cdot +C_{6}F_{6}\to C_{6}F_{5}+\text{HF}.$$ (24)

The radiation received in the vapor is relatively unimportant, estimated at 16×10^20^ ev as compared with 554×10^20^ ev in the liquid. In the liquid, the H_2_/C_6_F_6_ mole ratio is about 0.01, as against much higher ratios in the mixtures with C_6_H_6_ and C_6_H_12_. For comparable HF production in the two cases this would require that *k*_16_ be considerably greater than *k*_22_. For reactions (16) and (22) the activation energies may be near 6 kcal/mole or less. Somewhat analogous reactions with chlorine are:

$$\text{Cl}\cdot +H_{2}\to \text{HCl}+H\cdot ;E\approx 6\;\text{kcal}\;[41],$$ (25)

$$\text{Cl}\cdot +{\text{CH}}_{4}\to {\text{CH}}_{3}\cdot +\text{HCl};E<8\;\text{kcal}\;[42].$$ (26)

Of the other reactions, (17) should be discounted because of the failure to find C_6_F_5_H experimentally. Activation energies for reactions related to (17) and (18) have been estimated [28]:

$${\text{CF}}_{3}\cdot +\text{RH}\to {\text{CF}}_{3}H+R\cdot .$$ (27)

For the various hydrocarbons RH the activation energies of the reaction in kilocalories per mole are C_2_H_6_, 7.5; C_6_H_6_, 7.7; H_2_, 8.8; and CH_4_, 10.3. Although the basis of the estimates has been criticized [20], comparable work [23 to 29] is consistent with values somewhere near these. The abstraction reactions are generally expected to be slower than the additions to C_6_F_6_, as discussed earlier for pure C_6_F_6_.

The formation of HF can also be accounted for by excited-state mechanisms such as in reactions (20) and (21); there is, unfortunately, no explicit literature for comparison. The attempt to account for HF without C_6_F_5_H remains somewhat unconvincing, as reaction (21) could be followed by a combination of C_6_F_5_ and H.

### 3.3. Hexafluorobenzene and Benzene

The data for these mixtures are shown in tables 2 and 3. For comparison, results on C_6_H_6_ are reproduced from the work of other investigators [17]. The polymer analyses are low by 4.3 to 4.9 percent. Since fluorine analyses tend to be low, it is assumed for calculation that the true fluorine value is that obtained from the difference between (C plus H) and 100. Some of the deficit may, however, have arisen from oxidation of the polymer prior to analysis, which could have lowered the C, F, and H, and simultaneously introduced O and some H. To arrive at *G*(polymer) values, it was assumed formally that polymer is produced by withdrawing *x* moles of C_6_F_6_ and *y* moles of C_6_H_6_ from the liquid and rejecting *z* moles of HF. If H and F are lost in other forms and unequal amounts, small inconsistencies arise. On the basis proposed, one can calculate yields of each type of unit in the polymer and of HF lost per unit.

$$\begin{array}{l} \text{Base moles polymer}=x+y=\frac{\text{moles C}}{6}=\frac{\%C\times \text{weight}}{100\times 6\times 12},\\ \frac{\text{Moles HF lost}}{\text{Base mole polymer}}=z=3\left\{\frac{\text{moles C}-\text{moles F}-\text{moles H}}{\text{moles C}}\right\},\\ \frac{{\text{Moles C}}_{6}F_{6}}{\text{Base mole polymer}}=\frac{x}{x+y}=\frac{1}{2}\left\{\frac{\text{moles C}+\text{moles F}-\text{moles H}}{\text{moles C}}\right\}. \end{array}$$

**Figure 2:**
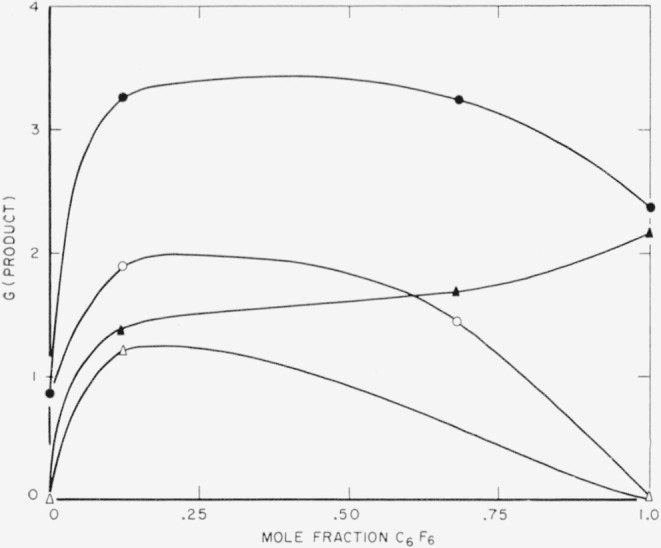
Radiation yields from *C_6_F_6_+C_6_H_6_*. ●, *G*(polymer, total), ○, *G*(polymer C_6_H_6_ units). ▲, *G*(polymer C_6_F_6_ units), Δ, 4*G*(SiF_4_).

For all the mixtures, *G*(polymer) is higher than for either pure component, and *G*(SiF_4_) is very much larger than in pure C_6_F_6_ (see fig. 2). The SiF_4_ was almost certainly formed from HF. The color of the polymer solution became darker with increasing C_6_H_6_ content, and the polymer during frozen benzene evaporation remained stiffer and spongier, never collapsing to a clear glass.

**Figure 3:**
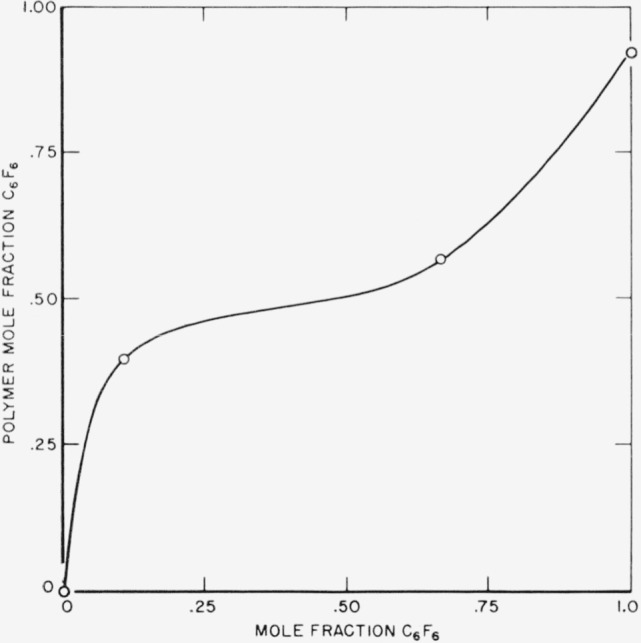
Composition of polymer from *C_6_F_6_+C_6_H_6_*.

The composition of the polymer remained near 1:1 for C_6_H_6_/C_6_F_6_, even for wide variations in feed ratio (table 2, fig. 3). At increasing C_6_H_6_ feed content, the H/C and F/C ratios of polymer gradually declined, reflecting the increased removal of H and F mentioned in connection with the SiF_4_ yields. As with the pure C_6_F_6_, the mixture did not show any CF_4_ or C_2_F_2_, and only the mixture with the high C_6_H_6_ content, 0.886 mole fraction, showed CH_4_ or C_2_H_2_. Surprisingly, neither C_6_F_5_H nor C_6_H_5_F was found.

The behavior of polymer composition is reminiscent of vinyl copolymerization with a strong alternating tendency. By analogy, a mechanism can be written in which large “crossed” propagation rate constants are responsible for the composition, i.e., where k_29_, k_31_>k_28_, k_30_.

$$〰C_{6}F_{6}\cdot +C_{6}F_{6}\underset{\to }{k_{28}}C_{6}F_{6}-C_{6}F_{6}\cdot ,$$ (28)

$$〰C_{6}F_{6}\cdot +C_{6}H_{6}\underset{\to }{k_{29}}C_{6}F_{6}-C_{6}H_{6}\cdot ,$$ (29)

$$〰C_{6}H_{6}\cdot +C_{6}H_{6}\underset{\to }{k_{30}}C_{6}H_{6}-C_{6}H_{6}\cdot ,$$ (30)

$$〰C_{6}H_{6}\cdot +C_{6}F_{6}\underset{\to }{k_{31}}C_{6}H_{6}-C_{6}F_{6}\cdot .$$ (31)

Polarity differences often favor addition of unlike units. In partial support, Szwarc [21] finds the methyl affinities of C_6_F_6_ and C_2_F_4_ to be 14 and 10 times greater than the methyl affinities of C_6_H_6_ and C_2_H_4_. The corresponding CF_3_. affinities do not seem to be known. For these low-molecular weight polymers, favorable crossed terminations could also influence the polymer composition.

There are several possible mechanisms involving excited states. Formally, a “crossed” generation of excited molecules could be favored by an energy transfer mechanism [43]. Against this concept is the fact that the overall formation of polymer is nearly independent of changing composition. It has also been pointed out in criticism that a symmetrical mutual energy transfer should not occur very generally [40]. Thus, an initial formation of triplet excited states or radicals of the two species in equivalent amounts by mutual transfer of excitation is unlikely. However, the chemical reactivity of triplet excited states may depend on some of the same considerations which apply to free radicals, among which polarity differences are included.

As in the other cases, a decision between the triplet state and radical mechanism is difficult. The obvious qualitative difference is that molecules are dissociated into fragments when forming radicals but not when forming excited states. The presence or absence of fragments is thus one criterion for distinction. Aside from this, it may be necessary to depend upon highly detailed knowledge of the two types of intermediates. Here, the formation of HF is weak evidence for the presence of some free radicals, while the absence of C_6_H_5_F and C_6_F_5_H is evidence against dissociation. A possible nonradical source of HF is from the reaction of excited molecules with other excited or ground-state molecules:

$$C_{6}H_{6}*+C_{6}F_{6}\to C_{6}H_{5}-C_{6}F_{5}+\text{HF}.$$ (32)

At low conversions, there are many more C_6_ molecules than any other species. The fact that the polymer formed is mainly higher than C_12_ indicates that the C_12_ species once formed must retain chemical activity. A radical-addition mechanism allows this to occur in a self-evident way:

$$C_{6}H_{5}\cdot +C_{6}F_{6}\to C_{6}H_{5}-C_{6}F_{6}.$$ (33)

In a pure triplet-state mechanism, it is not evident that the C_12_ species would remain in an excited state with a long lifetime. Possibly excited C_6_ could transfer excitation preferentially to ground-state C_12_ molecules. Both the relative absence of fragments and the growth of larger species could be explained if excited molecules initially combine to form a biradical which subsequently grows by ordinary radical addition.

The high *G*(HF) in mixtures could be formally accounted for by a Bagdassarian [43] or Magat [40] excitation mechanism. An atom and radical mechanism can account for this feature with emphasis on the steps:

$$C_{6}F_{6}\to C_{6}F_{5}\cdot +F\cdot ,$$ (6)

$$F\cdot +C_{6}H_{6}\to C_{6}H_{5}\cdot +\text{HF},$$ (22)

$$F\cdot +C_{6}H_{6}\to C_{6}H_{6}F\cdot ,$$ (34)

$$F\cdot +C_{6}F_{6}\to C_{6}F_{7}\cdot .$$ (7)

Here k_22_<k_7_<k_34_, but all are of substantial magnitude. To illustrate, a calculation with k_7_=0.1 k_34_, k_22_ = 0.05 k_7_ predicts a rather flat maximum production of HF near 0.7 mole fraction C_6_F_6_.

The data for the mixtures of C_6_F_6_ and C_6_H_6_ at 218° C are very rough because of the small sample size and difficulties of manipulation (see table 4). As with pure C_6_F_6_ at this temperature, the polymer was insoluble and dark. The *G* values for both polymer and volatile products seem to vary linearly with composition.

It is not surprising that *G*(SiF_4_) from C_6_F_6_ should be higher at high temperature (compare tables 3 and 4), but it is difficult to understand why *G*(SiF_4_) from the mixtures is less than at room temperature. The polymer from the mixtures resembles that from C_6_F_6_ at this temperature, and is presumably rich in condensed ring structures or conjugated unsaturation, the formation of which requires elimination of F_2_ or HF. Thus, the *G*(SiF_4_) suggests no change from mixtures at 20° C, while the insolubility and color suggest more HF elimination. The dark color of the polymer from mixtures does suggest some conjugated unsaturation even at 20° C. The principal remaining anomaly may then be the relatively high *G*(SiF_4_) from pure C_6_F_6_ at 218° C. This could be attributed to union of F atoms as to more efficient escape from a C_6_F_6_ cage to the wall, or to the onset of C—C cleavage like that which produced C_2_H_2_ from benzene. The C_2_F_2_ and similar fragments could be reactive with the walls of the apparatus.

### 3.4. Hexafluorobenzene and Cyclohexane

From C_6_F_6_ and cyclohexane, the principal products were SiF_4_ (*G*=0.322), H_2_ (*G*=1.92), and polymer (*G*= 3 to 5) (see table 6). The *G* value for the SiF_4_ is not very different from that of C_6_F_6_ and benzene. The polymer composition can be discussed by an extension of the method used for C_6_F_6_ and C_6_H_6_, if C–C chain scission products can be neglected. Let each C_6_ unit of polymer be build from *f* moles of C_6_F_6_ and (1–*f*) moles of C_6_H_12_, rejecting *i* atoms of F and *j* atoms of H. Then

$$\begin{array}{l} f=\frac{F}{C}+\frac{i}{6},\\ f\geq \frac{F}{C}\\ f=1-\frac{1}{2}\frac{H}{C}-\frac{j}{12},\\ f\leq 1-\frac{1}{2}\frac{H}{C}. \end{array}$$

Similar limiting formulas can be constructed for other systems.

The *G* value for H_2_(1.92) plus that derived for HF (1.29) is a little less than the usual *G*(H_2_) [44] for cyclohexane multiplied by the electron fraction (5.5×0.636=3.5). This may correspond to a weak protective effect. Of the H_2_, some may be formed by a molecular mechanism, but the HF should be from an abstraction by either of the following reactions:

$$F\cdot +C_{6}H_{12}\to \text{HF}+C_{6}H_{11}\cdot ,$$ (35)

$$H\cdot +C_{6}F_{6}\to \text{HF}+C_{6}F_{5}\cdot .$$ (24)

As discussed earlier for hexafluorobenzene and hydrogen, the evidence for reaction (24) or any abstraction from C_6_F_6_ is weak; for example, in the failure to detect C_6_F_5_H here. For the same reasons, radicals from C_6_H_12_ are not likely to abstract fluorine to form alkyl and cyclohexyl fluorides. The failure to find RF is not quite conclusive because C_6_H_11_F is rather unstable and smaller scission products of any one kind would be small in amount. On the whole, reaction (35) is preferred as the source of HF. The mechanism of protection may still be either of the sponge type or by addition, as with C_6_H_6_.

### 3.5. Perfluoroheptane

The data of table 7 are rather uncertain because of the large number of possible products and the general ambiguity of mass spectrometer analyses for saturated fluorocarbons. All of these compounds furnish large amounts of fragment ions, especially CF_3_^+^, and little parent ion. However, the *G* values for SiF_4_, CF_4_, and perhaps C_2_F_6_ are more reliable, as these components were determined at −80°, where most of the higher fluorocarbons are not volatile enough to interfere. The reported *G* value may, however, be low because of differential solubility of gases in the liquid at −80°; by contrast, both C_6_F_6_ and C_6_H_6_ have high freezing points and at −80° would have crystallized, expelling dissolved gases. The character of the polymer was similar to that reported by other workers. Most boiled in the range 179° to 250°, and the material was a viscous liquid at 4° C. For comparison, *n—*C_12_F_26_ boils at 175° and freezes at 42°, and *n—*C_16_F_34_ boils at 240° and freezes at 115°. The failure of the sample to freeze may indicate branched structures, but is not very conclusive in a mixture.

In addition to the data of table 7, the analyses of liquid in table 8 are of qualitative interest, although both the amounts and identities quoted are subject to large uncertainties of interpretation. Presumably, fractionation effects would weight unduly the lowest boiling components and minimize those above C_7_. The values of *G*(CF_4_) = 0.195 and of *G*(SiF_4_) = 0.167 for C_7_F_16_ are high relative to those for C_6_F_6_ (table 7). The main products are thus SiF_4_ and higher and lower saturated fluorocarbons. The mass spectrometer finds almost no olefinic molecules. It is possible that more sensitive and reliable indications would be given by infrared or by bromine or permanganate titrations. Stoichiometry requires that the extra fluorine content of the C_1_ to C_6_ perfluoroparaffins and the loss to SiF_4_ be compensated either by equivalent condensation to higher perfluoroparaffins or formation of double bonds. The following reactions are to be considered, where rates can vary with the size of the radicals *R_i_* and *R_j_*:

C—C scission:
  $$C_{7}F_{16}\to R_{i}\cdot +R_{7-i}\cdot .$$ (36)
C—F atom splitting:
  $$C_{7}F_{16}\to sec–C_{7}F_{15}\cdot +F\cdot ,$$ (37)
  $$C_{7}F_{1}\to prim–C_{7}F_{15}\cdot +F\cdot .$$ (38)
Recombination:
  $$F\cdot +F\cdot \to F_{2},$$ (13)
  $$R_{i}\cdot +F\cdot \to R_{i}F,$$ (39)
  $$R_{i}\cdot +R_{j}\cdot \to R_{i}-R_{j}.$$ (40)
Disproportionation:
  $$R_{i}\cdot +R_{j}\cdot \to C_{i}F_{2i}+C_{j}F_{2j+2},$$ (41)
  $$F\cdot +R_{i}\cdot \to C_{i}F_{2i}+F_{2}.$$ (42)
Radical attack on F_2_:
  $$R_{i}\cdot +F_{2}\to R_{i}F+F\cdot .$$ (43)
Transfer:
  $$R_{i}\cdot +C_{7}F_{16}\to C_{7}F_{15}\cdot +R_{i}F.$$ (44)

The transfer reaction (44) is unlikely because of energetics, as previously discussed. Disproportionation (41) and (42) would generate olefinic molecules and so should be unimportant here; moreover, there is convincing experimental evidence against reaction (41) [23 to 29]. The remaining reactions, coupled with material balance, all require conservation of total number of molecules, and thus a compensation between product molecules larger and smaller than C_7_. Larger molecules were found in the distillation residue although not by the mass spectrometer.

To extend the conclusion about conservation of number of molecules from C_7_F_16_ to high polymers appears inconsistent with the familiar rapid degradation of polytetrafluoroethylene and polychlorotrifluoroethylcne. The difficulty could be met by asserting that sufficient oxygen or hydrocarbon material was present in all polymer experiments to combine or undergo H abstraction with the polymer radicals and thus lower the molecular weight; or that the radicals were trapped in the solid matrix and could then undergo various other reactions ordinarily of low probability; or that a relatively very small increase in the number of molecules and double bonds occurs in all cases but is easily noticed only in the polymer. In this connection it has been noticed recently that polytetrafluoroethylene irradiated in vacuo is degraded very much less rapidly than in air [45]. Nevertheless, a thorough search for olefinic molecules in irradiated perfluoroheptane would be desirable.

The set of possible reactions is still too complicated for easy treatment, even when simplified by assumptions such as random splitting and equal reactivity of all radicals. Substantial amounts of all perfluoroparaffins C_1_ to C_14_ could be expected. By assuming that all C—C and C—F bonds split with equal probability, and that all lower radicals disappear only by reaction with F atoms with equal rate constants, one arrives at an initial fragment distribution:

$$F:prim—C_{7}:sec—C_{7}:\text{all}\;\text{lower}\;\text{alkyl}:{\text{CF}}_{3}=16:6:10:12:2$$

and a product distribution in which all lower perfluoroalkanes occur equally and *G*(CF_4_) = 2/46 times the *G* value of total initial atoms and radicals. The observed *G*(CF_4_) = 0.195 might thus indicate an initial *G* value for total atoms and radicals as high as 4.5.

In a liquid phase, the products will be governed by the chances for diffusion before recombination of fragments. The predominant reactions will thus be

$$C_{7}F_{16}\to C_{7}F_{15}\cdot +F.,$$ (37, 38)

$$C_{7}F_{16}\to C_{6}F_{13}\cdot +{\text{CF}}_{3}\cdot \left(\text{for}\;i=1\right)$$ (36a)

$$F\cdot +F\cdot \to F_{2},$$ (13)

$$F\cdot +{\text{CF}}_{3}\cdot \to {\text{CF}}_{4}\;\left(\text{for}\;i=1\right),$$ (39a)

$$2C_{7}F_{15}\cdot \to C_{14}F_{30}[\;\left(\text{for}\;i,j=7\right),$$ (40a)

$$2C_{6}F_{13}\cdot \to C_{12}F_{26}\;\left(\text{for}\;i,j=6\right).$$ (41a)

It can be seen that these reactions account for much of the product SiF_4_, CF_4_, and high-boiling residue.

### 3.6. Perfluoroheptane and Benzene

The mixture of perfluoroheptane and benzene was heterogeneous but of considerable qualitative interest. The principal products from irradiation were SiF_4_ (at a *G* value about twice that of pure C_7_F_16_), polymer, numerous lower fluorocarbons, and CF_3_H (table 9). The last named compound had a *G* value of 0.158 by calculation from analyses at −80°C; however, the sample, when later warmed to 25° C, contained large amounts of CF_3_H in the vapor phase, and a rough estimate from the 25° C analysis suggested a *G*(CF_3_H) in the region of 2.

Inasmuch as CF_3_H boils at −84°, and C_7_F_16_, the major component of the mixture, is sometimes liquid at −80°, it is not unreasonable that most of the CF_3_H present at −80° would be held in solution in the C_7_F_16_. For the same reason, *G* values for C_2_F_6_ and C_2_F_4_ should probably also be much higher. The CF_3_H indicates a clear-cut abstraction reaction from C_6_H_6_:

$${\text{CF}}_{3}\cdot +C_{6}H_{6}\to {\text{CF}}_{3}H+C_{6}H_{5}.$$ (45)

It is obvious from the presence of CF_4_, C_2_F_6_, etc., that the direct recombinations, F·+R· and R· + R·, still occur to some extent. A comparison with column 1 of table 7, especially the fairly reliable CF_4_ values, may indicate essentially no protection of C_7_F_16_ by either C_6_F_6_ or C_6_H_6_. The SiF_4_ values of table 9 indicate a sensitization, but the solubility of SiF_4_ in C_7_F_16_ under the conditions of analysis complicates the result, and both *G* values must be used with caution. Although C_7_F_16_ in benzene had only moderate *G* values for products, C_7_F_16_ in styrene was found to promote polymerization with a partial electron-fraction *G* value of 20±10[46]. The present C_7_F_16_ environment should be in some ways similar.

If the high estimate of *G*(CF_3_H) of about 2 is correct for C_7_F_16_ in benzene, it may be consistent with the polymerization *G*= 20 for C_7_F_16_ in styrene, for the various modes of C—C and C—F scission can form many other radicals as well as the CF_3_. If the low value in table 8 is more nearly right, then the high results with styrene may require a special energy-transfer effect. In the radiolysis of mixtures of C_6_H_6_ with C_6_H_5_CF_3_, CF_4_, and chlorofluorocarbons, Feng [13, 14] has reported *G*(radicals) of the order of 1 by the DPPH disappearance method in contrast with the high *G*(radicals) usually observed for other halocarbons [40]. Although Feng’s CF_4_ effects were observed at unknown high dilution and the difference from pure C_6_H_6_ was close to the experimental error, his *G* values for C_6_H_5_CF_3_ and CF_4_ in any event were not large, and thus differ from the case of the chlorocarbons. Considering all the evidence, it seems best to suppose that our true *G*(CF_3_H) is considerably less than 2, that *G*(radicals) is usually low for fluorocarbons, and that the high *G*(radicals) = 20 for C_7_F_16_ in styrene polymerization may be due to special energy transfer effects, valid for styrene but not benzene.

It was further reported by Feng that the irradiation of CF_4_ and C_6_H_6_ produced C_6_H_5_F and C_6_H_5_CF_3_, detected by infrared, with *G* values rising to 1.5 [13, 14]. In the present mixture, C_6_H_5_F was not found, although its formation by combination of F + C_6_H_5_ is not unreasonable. In the present study, the C_6_H_5_F would have been associated with large proportions of unchanged liquid and perhaps not detected with high sensitivity. The reported high *G* value of C_6_H_5_F from so dilute a solution of CF_4_ is surprising. Possibly other compounds, such as polymer structures formed by addition, could have absorbed at C—H and C—F frequencies close to those of C_6_H_5_CF_3_ and C_6_H_5_F, with a very large absorption coefficient.

### 3.7. Perfluoroheptane and Cyclohexane

The mixtures of perfluoroheptane and cyclohexane were heterogeneous. The only data are those upon polymer, table 9, as all containers were broken by corrosion of glass seals, even after relatively small doses of the order of 70 Mr. Presumably the yield of HF was considerably higher than any reported in the tables. Comparison of the polymer yields in columns 4 and 5 suggest that *G*(polymer) declines with increased dose.

### 3.8. Perfluorobenzene and Perfluoroheptane

With perfluorobenzene and perfluoroheptane (table 7) there appears to be an initial rise in *G*(C_2_F_6_) as the C_6_F_6_ content is increased. This is believed to be an artifact due to solubility, as the freezing of C_6_F_6_ will concentrate the gaseous solutes in the remaining liquid and vapor. The data on SiF_4_ and CF_4_ are subject less strongly to the same kind of error, which may seriously underestimate these products where much C_7_F_16_ is present. There is at least no strong “protective” effect. Since F atoms should be present, it appears that they combine with each other and with aliphatic radicals more readily than they add to C_6_F_6_. On the other hand, a rapid addition of F to C_6_F_6_ is needed in the radical mechanism for C_6_F_6_ radiolysis to explain the low SiF_4_ yield. This inconsistency may call into question any purely radical mechanism for the radiolysis of C_6_F_6_ and favor a triplet-state mechanism there. A similar argument may apply to the radiolysis of C_6_H_6_, which exhibits a protective effect less drastic than might be expected from the radiolysis of the pure component. The very low hydrogen yields from pure C_6_H_6_ may be due mainly to failure to split off hydrogen atoms, rather than to rapid reaction with them. The yield of radicals from C_6_H_6_ detected by ordinary methods is somewhat low; *G*=0.33–0.89 by iodine [47] and DPPH disappearance [40]. Possibly it is also of about this same magnitude in mixtures where C_6_H_6_ exhibits a protective effect. The “sponge” mechanism of protection in C_6_H_6_ mixtures has been discussed recently in terms of relations between excited states [40].

The failure of protection in the present mixture suggests that protection, where it occurs, is of the sponge type and not due to extreme reactivity of the aromatic ring with atoms and radicals, and that the characteristics of aromatic radiation chemistry (considerable polymer, very little hydrogen or halogen) depend more upon reactions which proceed via triplet states than upon atom and radical reactions.

## 4. Conclusions

The data presented here show that representative pure liquid fluorocarbons are not especially sensitive toward ionizing radiation. In the paraffinic series, the indicated C—C scissions are about equal in *n*-C_7_F_16_ and *n*-C_7_H_16_, as judged by the respective *G*(CF_4_) and *G*(CH_4_); and the indicated C—F scissions of the fluorocarbon are much less than the C—H scissions of the hydrocarbon. The low yield of C—F scission products (SiF_4_) may be a cage effect phenomenon. The diffusion away of the hydrogen atom of a C—H pair must be an easier process than the corresponding diffusion of a fluorine atom. Results in the gas phase would be interesting for comparison Even less C—F scission than that found here is suggested by the fact that Simons and Taylor [5], irradiating perfluoroaliphatic compounds in all-alumiuum containers, found no evidence whatever of corrosive fluorine.

Aside from differences of purity or analytical sensitivity, both sets of observations appear consistent with the existence of a small steady-state concentration of F_2_, which disappeared in one instance by diffusion to the glass parts of the apparatus and conversion to SiF_4_, and in the other by attack of fluorocarbon radicals to form lower perfluoroalkanes. Some minimal C—F scission seems necessary to account for the considerable amount of C_13_ and C_14_ coupling products from C_7_F_16_. Irradiated polytetrafluoroethylene seems to undergo C—F scissions exclusively, according to electron resonance observations [48]. This behavior is again consistent with a cage effect, as F from a C—F scission can diffuse away, while the radical pair from a C—C scission is held more rigidly and recombines.

Some of the early indications of fluorocarbon sensitivity were due to the presence of oxygen. Recent studies of the tensile strength of irradiated polytetrafluoroethylene show the loss of tensile is very rapid in the presence of oxygen and hardly perceptible for long periods in its absence [45]. The strong oxygen effect is reminiscent of the degradation of very pure chlorinated compounds exposed to light, air, and moisture. For fluorocarbons under irradiation it may be speculated that the radical recombination rate is somewhat slower than for hydrocarbons, allowing more effective competition by oxygen reactions.

Aromatic fluorocarbons have the same kind of resistance to ionizing radiation as the aromatic hydrocarbons, yielding very little gas and a moderate amount of low polymer, *G*(polymer) is 2.01 for C_6_F_6_, as against 0.93 for C_6_H_6_. The polymers from both materials are close to the starting material in elemental analysis. There has been some speculation in the literature concerning the degree of aromatic character present in perhaloaromatic compounds [49]. At least those aspects of aromatic character concerned with radiation resistance seem to remain in the totally fluorinated analog.

Recalling the considerable resistance of polystyrene to radiation, one might predict a similar resistance in polymers containing perfluoroaromatic groups.

Experimentally, poly(2,3,4,5,6-pentafluorostyrene) has a *G* value for free radicals observed by electron spin resonance, almost as low as polystyrene itself [50], which suggest that the general radiation resistance might be similar. Studies on mechanical and solution properties of large samples would be of interest, as would studies on poly (perfluorostyrene) if it should become available. Presumably, polymers with perfluoroaromatic rings in the main chain, rather than a side chain, would show a better combined resistance to heat and radiation than any styrene derivative. Polyphenyls and perfluoro-polyphenylene ethers are the obvious structural possibilities of this kind.

It is not surprising that mixtures of fluorocarbons with hydrocarbons are usually less stable to radiation than the pure components themselves, for the production of hydrogen fluoride is now possible. It is likely that most partially fluorinated compounds would have the same weakness. In spite of the general tendency toward increased sensitivity, hexafluorobenzene appears to repress somewhat the production of hydrogen from cyclohexane. The increased polymer production in mixtures is in most cases a complicated phenomenon, but in the benzene-hexafluorobenzene mixtures it exhibits a strong tendency toward equal numbers of benzene and hexafluorobenzene units, as in alternating copolymerization. A likely reason for this behavior is the enhancement of radical or triplet-state reactivities by polarity differences.

At ordinary temperatures, atom and radical mechanisms modified by cage effects seem able to account for the results. Mechanisms involving triplet states, as outlined by other authors for benzene, are perhaps preferable for the perfluoroaromatic systems, especially because of the very slight occurrence of fragmentation. Ionic mechanisms, proposed by Feng for certain hydrocarbon fluorocarbon mixtures, have not been considered here at length because of the fairly satisfactory explanation by other mechanisms and the very short lifetimes to be expected for ions generally in condensed systems.

At higher temperatures (218° C), the radiation chemistry of hexafluorobenzene is not well understood, but the material retains a fairly good resistance toward inorganic fluoride production and the usual tendency for polymer production. Pure fluorocarbon materials are thus not especially sensitive to radiation, and aromatic fluorocarbons are quite resistant.

## Figures and Tables

**Table 1:** Radiation yields for several processes

Process	*G* value		
	C*_n_*H_2_*_n_*_+2_	C_6_H_6_	C*_n_*F_2_*_n_*_+2_
			
C—C scission	<1	0.02	1.7
H_2_ or F_2_	5	.04	.1
Polymer or crosslinks	5	1	0

**Table 2:** Polymer from irradiated hexafluorobenzene and benzene at 20° C^a^

C_6_F_6_ in feed, mole fraction	1.00	0.667	0.114
			
C_6_F_6_ in polymer, mole fraction	0.922	0.527	0.422
Polymer weight g	.7051	.6822	.860
%C	40.2	55.3	61.2
%H	b0.3	2.	2.7
%F^c^	55.	38.4	31.2
Base moles	.00394	.00524	.00731
HF moles lost per mole	0	.230	.296

a Exposure dose 275 Mr.

b Presumed absent from fresh polymer.

c For further calculations, F is taken as 100—C—H.

**Table 3:** Radiation yields from hexafluorobenzene and benzene at 20° C^a^

C_6_F_6_ in feed, mole fraction	1.00	0.667	0.114	0^c^
				
C_6_F_6_ in polymer, mole fraction	0.922	0.527	0.422	0
*G*(polymer)^b^	2.01	2.73	2.79	c.93
*G*(C_6_F_6_ units)	1.85	1.44	1.18	……….
*G*(HF lost)^d^	……….	0.628	.826	……….
4*G*(SiF_4_)^e^	.045	Present	1.334	……….
2*G*(H_2_)	.0014	Lost	.126	c.088

a Exposure dose 275 Mr; dose factors for C_6_H_6_, 0.589×10^20^ ev/g-Mr; for C_6_F_6_, 0.530×10^20^ ev/g-Mr.

b C_6_H_6_ and C_6_F_6_ units.

c From Gordon et al., ref. [17].

d From polymer analysis.

e From gas analysis.

**Table 4:** Radiation yields from hexafluorobenzene and benzene at 218° C^a^

C_6_F_6_ mole fraction	1.00	0.256	0
			
4*G*(SiF_4_)	0.84	0.105	0
*G*(CO)	.054	.0063	0
*G*(CO_2_)	.022	.0048	0
*G*(H_2_)	0	.0075	.0022
*G*(C_2_H_2_)	0	.0048	.017
*G*(polymer)^b^	1.3±0.5	1±0.5	1.3

a Exposure dose 350 Mr.

b C_6_H_6_ and C_6_F_6_ units.

**Table 5:** Radiation yields from hexafluorobenzene and hydrogen 20° C^a^

	C_6_F_6_	C_6_F_6_+H_2_
		
4*G*(SiF_4_)	0.045	0.440
*G*(CO_2_)	.004	.004
*G*(CO)	.0012	0
*G*(polymer)^b^	2.01	2.30

a Exposure dose 319 Mr; hydrogen pressure 34 atm.

b C_6_F_6_ units.

**Table 6:** Radiation yields from hexafluorobenzene and cyclohexane at 20° C^a^

C_6_F_6_, mole fraction	O^b^	0.192	0.234	0.655
				
4*G*(SiF_4_)	0	(c)	1.29	(c).
*G*(H_2_)	5.2, 5.9	(c)	1.92	(c).
*G*(CH_4_)	0.09,0.02	(c)	0.005	(c).
*G*(C_2_H_4_)	0.21,0.14	(c)	0.007	(c).
*G*(polymer)^d^	1.66^b^	4.9	6.1	3.1.
Polymer F/C^e^	0	0.336	0.368	0.479.
Polymer H/C^e^	………………	1.279	1.148	0.925.
Polymer, mole fraction C_6_F_6_	0	0.34 to 0.36	0.37 to 0.43	0.48 to 0.54.

a Exposure dose 174.5 Mr.

b Ref[19], pp. 18 and 20.

c Container failed before analysis.

d C_6_ units.

e Mole ratio.

**Table 7:** Radiation yields from hexafluorobenzene and perfluoroheptane at 20° C^a^

C_6_F_6_, mole fraction	0	0.38	0.74	1.00
				
4*G*(SiF_4_)	b0.668	1.232^b^	0.352	0.040
*G*(CO_2_)	b.028	0.025^b^	0.003	.0012
*G*(CF_4_)	.195	0.131	0.085	0
*G*(C_2_F_6_	b.081	0.122^b^	0.008	0
*G*(polymer)^c^	2.0	3.2 to 3.8	3.0 to 3.5	2.01
Polymer F/C^d^	2.102	1.624	1.361	.93
Polymer mole fraction C_2_F_6_.	0	<0.55	<0.75	1

a Exposure dose 330 to 408 Mr; dose factor for C_6_F_6_, 0.530×10^20^ ev/g Mr; for C_7_F_16_, 0.526.

b May be low because of solubility.

c As C_6_ or C7 units.

d Mole ratio.

**Table 8:** Liquid phase from radiolysis of hexafluorobenzene and perfluoroheptane^a^

C_7_F_16_ prepared mole fraction	1.00	0.62
C_7_F_16_ found mole %	9.9	19.3
C_6_F_6_ found mole %	0	9.3
C_6_F_14_ found mole %	7.0	10.2
C_5_F_12_ b found mole %	6.0	5.7
C_3_F_8_ found mole %	9.0	1.5
C_2_F_6_ found mole %	2.19	~0

a See table 7, footnote ^a^, for radiation conditions. Samples analyzed by mass spectra of vapor at 25° C.

b Or *cyclo*-C_5_H_10_.

**Table 9:** Radiation yields from hydrocarbon-perfluoroheptane mixtures at 20° C^a^

C_7_F_16_, mole fraction	0.716	0.560	0.685	0.188	0.213.
Second component	C_6_H_6_	C_6_H_6_	*c*-C_6_H_12_	*c*-C_6_H_12_	*c*-C_6_H_12_.
Dose Mr	339	174	339	339	174.
4*G*(SiF_4_)	(b)	2.62	(b)	(b)	(b).
*G*(H_2_)	……	0.09	……	……	
*G*(CF_3_H)	……	0.158^d^	……	……	
*G*(CF_4_)	……	0.189	……	……	
*G*(C_2_F_6_)	……	0.021^d^	……	……	
*G*(C_2_F_4_)	……	0.017^d^	……	……	
*G*(CH_4_)	……	0	……	……	
*G*(C_2_H_4_)	……	0	……	……	
*G*(polymer)^c^	4.2 to 4.9	5.8 to 6.8	2.7 to 3.1	2.8 to 3.2	4.7 to 5.4.^e^
Polymer H/C	0.340	0.305	0.866	1.326	
Polymer F/C	1.337	1.357	0.951	0.502	
Polymer, mole fraction C_7_F_16_	0.55 to 0.62.	0.56 to 0.66.	0.38 to 0.53.	0.19 to 0.3C.	

a All samples have two liquid phases.

b Large; failure through glass seal corrosion.

c In C_6_ or C_7_ units, from weight and carbon analysis.

d May be much too low; large content remains in room temperature analysis.

e From weight, assuming analysis of preceding column.

## Footnotes

2 Figures in brackets indicate the literature references at the end of this paper.

3 Combustion analyses were by E. R. Deardorff, NBS.

4 Spectra were obtained by John J. Comeford and W. J. Pummer, NBS.
